# Cursory Remarks on the Diagnosis of Fatty Heart

**Published:** 1859-10

**Authors:** Henry Kennedy

**Affiliations:** Fellow and Censor of the College of Physicians in Ireland, Physician Extraordinary to Sir P. Dun’s Hospital


*Cursory Remarks on the Diagnosis of Fatty Heart.* By Henry Kennedy, A.B., M.B., Fellow and Censor of the College of Physicians in Ireland, Physician Extraordinary to Sir P. Dun's Hospital. (Read before the Medical Association, June 1, 1859.)

(From the *North American Medico-Chir. Review.*)

The knowledge of morbid anatomy has increased much within the last twenty-five years; nor must we forget that Dublin has the credit of taking the first step in this great move, by the establishment of the Pathological Society; and a student can now, by very little trouble, attain a knowledge of morbid structure which would have been utterly impossible for even the oldest to acquire a few years since. Of the various paths to which the study of diseased structure has led, none has been more striking than the one known under the name of fatty degeneration; and very few are the parts of the frame in which this change has not been detected. It is not my intention here to speak of this subject generally. One remark, and only one of a general character, would I make; and this is, that the change known as fatty must be looked on in a different point of view from any other degeneration with which we are familiar. Neither malignant, strumous, nor other changes are to be found in the frame which is healthy. But fat is ever, and must ever be, a constituent part of it. Hence, when its presence amounts to disease, it is only an exaggeration of a healthy condition, if I may so speak. Hence, also,—and this is a deduction which it may be of much greater consequence to keep in mind,—the powers of art may fairly be looked on as more capable of grappling with this change than with others: of which more again.

I have said it is not my object to speak of fatty change generally. My present wish is to direct attention to a few points connected with this change when it affects the heart, and, more particularly, the diagnosis of this state. It will, I think, be admitted that this point is one of grave importance not only to the physician, but also the surgeon. You must be now well aware that all, or very nearly all, the cases of sudden death under the use of chloroform, have taken place in persons affected with fatty hearts. This fact alone would invest the subject with consequence; to say nothing of the interest it presents to the physician. It is, in fact, a common ground for both, and well worthy their deepest attention.

It is necessary to begin these remarks by avowing the great difficulty which surrounds the diagnosis of fatty heart. Of the common affections of the organ—and this change may fairly be classed among these—there is none which presents more; and though on a former occasion I have directed attention to this point, it is essential to do so, but very briefly, again. I believe here, as with other diseases difficult of diagnosis, that the very first step in advance consists in our getting a clear idea of the difficulties which must be surmounted. Why, then, are there difficulties in this particular affection, more that in others of the same organ? One of the reasons was given by myself so far back as the year 1849; and I would now state my impression that the fact is one of moment, as bearing directly on the point under discussion. In the course of some investigations made in that year, I was led to observe that fatty disease of the heart and disease of the valves are not usually found together. This was, at first, an impression; but as this may be erroneous, I set about collecting data on which to found solid conclusions, and in a short time had collected some 53 cases. Of these, I found the valves were healthy in 43, which left 10 only of an opposite character. Here, then, was a fact of some consequence; and which I have taken every opportunity of testing since, and with no other result than still further confirming the point. I have noted 152 additional cases, so that the entire now amounts to 205;* and of these the valves were diseased in only 30. These numbers, I think, speak for themselves, and appear to myself to prove that valvular disease and fatty heart are rarely co-existent. The character of disease of the valves too, even when it does exist, is worth nothing; for it is very generally, though not invariably, of the thickened leathery kind: in fact, it is fatty disease of the valves, as contradistinguished from what is called ossific deposit. And this fact, again, has an important bearing on the progress of these cases, though I am not aware it has been noticed at all; for even though the valves be thickened, they may still be quite competent to perform their functions, at least so well that the life of the patient will not be shortened. In such, a souffle may be present, and persist for years and years without change, its character all through being very soft, and in some I have seen, hard to catch. The character of the souffle then has, when present, an important bearing on the case.

But before saying more on this point, I would notice a second, and, as it appears to me, a most important reason, why the diagnosis of fatty heart is often so very obscure. I allude to the fact that this change may be, and often is, partial. Of the fact you are all aware, and know that a very small portion of the left ventricle may be quite degenerated, the remainder being comparatively sound; and that, when rupture occurs, it is much more commonly in the walls of this cavity, and in a spot where they are both degenerated and thinned. Now, from this point it follows that five-sixths of the ventricle may be quite fitted to carry on its functions, and yet the other sixth be in a state which renders it liable to give way, so causing instant death; and all this without any physical sign which would be available during the lifetime of the patient. Important as this consideration is, however, it is not in connection with partial degeneration of one

* In tabulating these cases, I found it impossible to separate those which have been properly called true fatty degeneration from the others. I believe, however, that when fat exists in unusual quantity on the heart, it is rare to find the muscular structure beneath perfectly healthy.

cavity to which I wish now to direct your attention, but to the same state when it affects one side of the organ differently from the other; that is, when the left heart is more degenerated than the right, or *vice versa*. That this state occurs is perfectly well known; but it may be questioned whether the deductions which arise directly from it have received that attention, if any, which they appear to me to merit. Indeed, I am not aware of any author who has dwelt on this special subject at all. As illustrating what I mean, attention may be directed to the fifty fatal cases, under the use of chloroform, detailed in the able work of the late Dr. Snow. Any one who has read these cases may recollect how pointedly it is stated, I believe in every one of them, that the pulse at the wrist was closely watched, and, in almost all, continued to beat steadily till the very instant of death. Now it is not to be denied that watching the pulse is essential in such cases. But it is fully as essential to recollect that the healthy beat of a radial pulse may not be, and often is not, a guide to the state of the right heart. Hence the latter may have its action fatally stopped, by such an agent as chloroform, at the very moment when the left ventricle is carrying on its functions, to all appearance, healthily. And the examination of all the fatal cases seems to lead to the same conclusion; for it will be recollected that fluid blood and greatly distended right cavities were found in very nearly every fatal case. In point of fact, we have no pulse to guide us as regards the right heart; and hence a most important assistance is wanting to aid our diagnosis. In following out this part of my subject, I have been able to collect only a comparatively few cases; for attention not having hitherto been directed to the point, by far the greater number of those who have detailed cases of fatty heart have drawn no distinction between the cases, as to whether the disease was farther advanced on the one side or the other, or both. As it is, Quain gives 22 instances where the point is explicitly stated. Of these, the left ventricle was most affected in 8 cases, and the right in 4. In the remainder, the disease was equally divided. But I would not lay any great stress on these numbers; for they are too few to draw safe conclusions from. Two points, however, they show clearly: that either side of the heart may be seriously damaged while the other is comparatively healthy; and, secondly, that the disease to which the right heart is by far the most liable is that of fatty change: I mean as contrasted with valvular disease.

For so far, then, your attention has been mainly drawn to two points: first, that fatty degeneration and valvular disease rarely coexist; and, secondly, that the fatty change may be in great part limited to one or other side of the heart. But facts like these are useless, unless they lead to some practical results. Let me, then, draw some conclusions which appear to myself to follow directly from them.

Dr. Stokes, in his last able work, speaking of the diagnosis of fatty heart, lays down the following proposition: "That although this affection may exist without valvular disease, yet the coexistence of a certain amount of alteration of the aortic valves is common; so that the combination of a slow pulse, a feeble impulse, and a diminished first sound over the left ventricle, attended with a single murmur, while the second sound remains clear, will be sufficient for the diagnosis of the disease in many cases." Now, I believe this proposition to be correct as far as it applies; and it is certainly interesting to observe that it bears out curiously, by examinations on the living, the state of morbid parts, to which your attention has been directed this evening. For you will recollect it was stated that when valvular disease exists with fatty heart, the valves were what might be called leathery; that is, a state which might give rise to a murmur with the first sound, and yet leave the second healthy; and my own examinations on the dead body showed me that the valves, in this state, were quite competent to prevent all regurgitation, and thus give a healthy second sound. The proposition, I have said, was correct as far as it applies; but after the numbers given this evening, it is obvious it must be taken as proving the exceptional case. For they show, to my own mind indisputably, that fatty heart is much more commonly found existing by itself than with valvular disease. My first paper on the subject appeared five years before Dr. Stokes's work, and I take it for granted must have escaped his notice; otherwise I think his views would have been modified by it.

In connection with this thickened state of the valves, as contrasted with ossific deposit, there is another point worthy of notice. It has been long usual to connect visible pulsations of the arteries with open aortic valves. I satisfied myself, many years back, that this statement must be received with exceptions. In other words, you may have visible bounding of the arteries, where the valves of the aorta are not open, and where they prevent any regurgitation; and it is, as far as I am aware, when the valves are partially thickened that this state of things is to be observed. In such cases you may have a single first sound, as observed by Dr. Stokes; but in addition you may have—for it is not constant—visible pulsation of the arteries. And it need scarcely be observed that this bears, in the most direct way, on our prognosis; for this pulsation is not of the serious character of the disease described so well by Dr. Corrigan, and, I know from observation, does not affect the patient's life in the same way, and may go on unchanged for years. This thickened, and, as I believe it may be called, fatty state of the aortic valves, it is then essential to distinguish from any other diseased state of the same parts. It does not, I repeat, affect life in the same way as other valvular disease; is found chiefly in connection with fatty change in the texture of the heart itself; gives rise, in some instances, to visible bounding of the arteries, but at the same time not allowing of regurgitation; and, if to Dr. Stokes's observations my own be added, may, I believe, be diagnosed during life.

We now pass on to a consideration of those cases of fatty heart where no valvular disease exists; and here it is, in truth, that the diagnosis of fatty heart really commences; in other words, in a considerable majority of cases, no morbid sound exists by which our attention might be directed to the organ. If there be a morbid sound of any kind, it must lead to further investigation. But in its absence matters become very different. Hence we must start with the definite idea, that the great probability is, no morbid sound will be present to assist us. But, before going further, I would call attention for a moment to one or two points, which, in an inquiry of this kind, may not be out of place.

In the first place, I would notice the very peculiar arrangement of the muscular structure of the organ. A boiled heart is an excellent way of showing the very extraordinary way in which the muscles are interlaced with each other, and yet at the same time so disposed of in layers one over the other, that they might be described as being separate. To this state of parts we must add the peculiar arterial supply; for the coronary arteries barely, if at all, inosculate. In the large nervous supply, too, we must not overlook the close connection of the heart with the system by means of the eighth pair, at the same time that it receives a large supply from the sympathetic, and has, so to say, a special supply within itself, in the existence of ganglions found in its substance, and discovered, I believe, by Lee. In the last place may be noticed the way in which fatty disease exhibits itself in this organ. It is often, as all are aware, in spots which may be wonderfully isolated, being surrounded by healthy structure; or, it may be in layers, giving a striated appearance to a section of the walls of the particular cavity affected; or, should the entire thickness of the part be attacked, it is then usual for the inner surface to be the most diseased; or, with the muscular structure healthy, fat may be deposited on the surface of the organ in such quantity as to impede its action, and so constitute disease.

Keeping now these facts, both of the healthy and morbid heart, in view, on what, then, is the diagnosis of the disease to be founded? And it is to be remembered that you have no abnormal sound to assist you. Attention must be caught if there be any unusual murmur. But the existence of murmur is the exceptional case. After a good deal of attention to the subject, I really see no means of making a diagnosis, except, after the French method, by way of exclusion; and it even is not all that can be desired. I am well aware that writers give several physical signs as diagnostic, such as a weak beat at the heart or of the pulse, or muffled, distant or weak sounds. But I have the strongest conviction on my mind that, in so doing, they have not made allowance for what obtains in health. They have argued as if the impulse, sounds, and pulse were, so to say, fixed quantities; which I believe to be an error. For I have satisfied myself—as others, I think, must have done—that, within certain limits, and in a state of perfect health, both the sounds and the impulse of the heart vary very considerably; and so it is that you may have a strong or a weak pulse or impulse, and a great variety in the intensity of the sounds of the heart; and all these, I repeat, within the circle of health. The very same remarks, too, apply to the respiration, as I am sure all present must have observed. If, indeed, we see a case where, after the sounds have been natural, they then become muffled or weak, such would be a source of diagnosis. But every one is aware that cases do not come under notice, and that our opinion must very usually be formed from one or two observations. Should the disease be in the very advanced stage, then also, from the extreme feebleness of the sounds and impulse, assistance is afforded us. But at this period there are other signs which clearly show the state of the organ; and I would speak of an earlier stage, when it is really of much greater moment to make out the disease.

For the reasons just given, I would then say that in the comparatively early stage of fatty heart, we must look with great caution to either impulse or sounds to assist the diagnosis, unless in the exceptional case of which I have spoken. There is a physical sign, however, which, though not always present, may give us valuable assistance. Nor do I find that any writer has noticed it in connection with the diagnosis. I speak of enlargement of the heart where there is also fatty degeneration, and this without valvular disease. In Quain's valuable tables, which I take as independent observations, and so going to confirm my own, out of 83 more than half presented this state. Hence I cannot but conclude that enlarged heart forms an important element in what may be called the natural history of the affection; and I do not find that, in this point of view, it has been noticed by any recent writer. Fatty change is, in truth, by far the most frequent cause of enlargement of the organ, where there exists at the same time no valvular disease. For every one must be aware that hypertrophy *per se*—the heart's texture remaining sound—is very rarely met with. Hence, if we have an enlarged heart and healthy valves, the strong presumption is that the organ is fatty. But even with a previous knowledge of this fact, I am ready to admit that enlargement, even to double the average size, is not by any means so easily made out as might *a priori* be supposed. In the great majority of the cases, there is more fat than natural under the skin; and nothing is more common than to find it in quantity in the anterior mediastinum, to say nothing of the varieties to be met naturally as regards the relative position of the lung and heart. Still the difficulties may, I know, be often overcome by an accurate examination; and I would specially mention the plan of using percussion in varied positions of the body. But some one may ask here, is not this enlargement of the organ just as likely to be overlooked as any other physical sign connected with the disease? My answer is in the negative; and for this reason, that the pulse at the wrist leads us to look for it. You are all aware that on this last sign much has been written as diagnostic of fatty heart. I must here state my conviction, that it has been the exceptional cases which have been commonly described as marking the disease; such as slow, unequal, intermitting, or very rapid pulse. Now, I do not deny that these occur in cases of fatty heart. I have met examples of all of them. But what I do say is this: that the pulse most usually met is natural as to frequency; at the same time that it is fuller, passing sedately, as it were, under the finger, and giving the idea of being diffluent. It is, in truth—contrary to what is usually taught—the pulse of hypertrophy, but not attended with the same strength; and Hunter's expression might almost be applied to it, as being "action without power." Now, you will observe that such a pulse is exactly in keeping with the state of the heart which has been just described; it is what we would expect; and whatever doubts may arise about the most common character of pulse, there can be none, it may be stated, as to the frequency with which enlargement of the heart occurs in fatty degeneration, being fully one-half the cases detailed. I stop not to inquire the precise cause of the hypertrophy, but only to state the fact, and my own experience about it.*

Of the other kinds of pulse met in these cases I have little to say. Next in frequency to what has been described I would place inequality of the beats, not intermission. Some writers, I observe, speaking of this sign, seem at a loss to account for it, in the absence of valvular disease. To myself a fair explanation is afforded in the peculiar anatomy of the organ, glanced at this evening, taken in connection with the fact that fatty disease exhibits itself in spots, patches, or layers; a state of parts which may, without any straining, be expected to give rise to unequal action of the organ, and, consequently, unequal pulse: and, indeed, I may say that I have seen cases where a kind of double beat at the heart was attended by but a single one at the wrist.

Of the several symptoms in connection with fatty heart, and referred to the head, chest, or abdomen, it is not my intention here to speak. I would refer to the several writers on the subject; but more particularly to the able work of Dr. Stokes, which, as far as I am aware, contains the best *resume* of the matter. There are just two symptoms which appear to me to call for a moment's notice. The first is the dyspnœa, which, you are all aware, constitutes such a striking feature of the disease, especially in the middle and latter stages. Now, it is not the symptom itself to which I would direct attention, but the disproportion which almost constantly exists between the complaint of the patient and the labor of the respiration as evidenced to our eyes. In the history of the complaint, I am really not aware of any other single symptom more striking than this. The patients tell you that their great suffering is from dyspnœa, that they cannot make the slightest exertion; and yet, when you look at them, you see little or no corresponding movement of the chest. The contrast between this state and where there exists valvular disease, as, for instance, of the left auriculo-ventricular opening, is truly striking. Nor does this state exist only where there is fatty heart. It will be observed where the patient, in addition, gets an attack of bron-

---

* It has yet to be determined whether slow pulse occurs in any particular form of fatty heart. That it is by far the exceptional case, I have no doubt; and were I to venture to connect it with any one state of the organ, it would be that in which, without any enlargement, there existed true degeneration of the heart's texture; the patients being anything but fat. In most of such cases the effects of wine are truly remarkable.

chitis, very slight to all appearance, and yet a most fatal affection; and I have reasons for knowing that grave errors in prognosis have arisen in these cases, as, I believe, from the very cause to which I allude; that is, the respiration looks so quiet as to throw the medical man off his guard. It was gradually that I arrived at what appear to be fair reasons for this state of things. In the first place, the cartilages of the ribs are very generally more or less ossified; in the second, the intercostal muscles are very apt to be either covered with fat or degenerated; and, in the last place, the heart itself, while seriously altered in its texture, does not present any impediments, in the shape of valvular disease, to the current of the blood; and thus it is, with a disease of slow progress, as we must suppose fatty degeneration to be, that the system gets gradually accustomed to the change, and to a degree which it is impossible for it to do when there exists a direct obstacle to the circulation. It has further appeared to me that this want of proportion—as it may be well called—between the patient's sufferings and the physical exertion to relieve them, is particularly to be observed when the right heart is the furthest advanced in disease; and bearing out this, I have seen cases where the pulse at the wrist was steady and equal, not beating more than 86 in the minute, and this going on to the very last moment of existence. At any rate, whatever the explanation be, the fact itself has repeatedly come under my notice, and appears to me one well worthy of attention.

In the last place, I would notice a general sign which I think of consequence to look for, even though not always present. I mean a failure in the animal heat. It has been noticed casually by some writers. I would be inclined to give it much greater prominence. In some cases I have seen it was very striking; and it is worthy of note, as not being confined to the extremities alone. The patients have complained of it in the sides, and back, and stomach. Any complaint of this sort should ever, I believe, catch our attention.

It was my intention to have said something on the treatment; but my present limits forbid. I shall, therefore, only observe, that I was glad to find the possibility of a cure in the early stage of fatty heart, which was strongly advanced by myself ten years ago, and since then been supported by Dr. Stokes, in opposition to the views of Quain and others. But I can only here remark, that if we should attempt the cure, we would not have, in the majority of cases, any valvular disease to contend with.

In concluding these remarks, which it must be recollected are strictly cursory, it may be well to throw into a series of propositions the chief points brought forward.

1. That fatty change in the heart is rarely attended with valvular disease.

2. That, with our present knowledge, the proportion seems to be as six to one.

3. That when valvular disease exists with fatty heart, it is commonly the aortic valves that are affected, and these are thickened and fatty.

4. That this state of the valves rarely allows of regurgitations.

5. That it may give rise to a soft souffle with the first sound of the heart, leaving the second healthy, as observed by Dr. Stokes.

6. That there are grounds for supposing that this fatty state of the aortic valves may go on for years, without affecting the duration of life.

7. That visible pulsation of the arteries often attends this state; but as there is no regurgitation, it so differs from the disease described by Dr. Corrigan.

8. That enlargement occurs in more than half the cases of fatty heart.

9. That, in keeping with this, a large diffluent pulse is the most common kind to meet in fatty heart.

10. That either a very slow, unequal, or rapid pulse is only met in exceptional cases.

11. That the French way of exclusion is, in the absence of valvular disease, the chief way of arriving at the diagnosis of fatty heart.

12. That there is often a marked disproportion between the complaints of the patient of dyspnœa, and the physical efforts made to relieve it.

13. That this is possibly most marked when the right heart is the furthest advanced in disease.

14. That a marked diminution of the animal heat, which may, too, be confined to unusual parts of the body, is often met in connection with fatty degeneration.

15. That fatty degeneration of the right heart is the disease which most frequently affects that portion of the organ.—(*Edinburgh Medical Journal*, July, 1859.)

